# Raman Evidence of p53-DBD Disorder Decrease upon Interaction with the Anticancer Protein Azurin

**DOI:** 10.3390/ijms20123078

**Published:** 2019-06-24

**Authors:** Sara Signorelli, Salvatore Cannistraro, Anna Rita Bizzarri

**Affiliations:** Biophysics & Nanoscience Centre, DEB, Università della Tuscia, 01100 Viterbo, Italy; signorellis@unitus.it (S.S.); bizzarri@unitus.it (A.R.B.)

**Keywords:** Raman spectroscopy, p53, intrinsically disordered protein, blue copper protein Azurin, protein–protein interaction, Amide I band deconvolution, principal component analysis

## Abstract

Raman spectroscopy, which is a suitable tool to elucidate the structural properties of intrinsically disordered proteins, was applied to investigate the changes in both the structure and the conformational heterogeneity of the DNA-binding domain (DBD) belonging to the intrinsically disordered protein p53 upon its binding to Azurin, an electron-transfer anticancer protein from *Pseudomonas aeruginosa*. The Raman spectra of the DBD and Azurin, isolated in solution or forming a complex, were analyzed by a combined analysis based on peak inspection, band convolution, and principal component analysis (PCA). In particular, our attention was focused on the Raman peaks of Tyrosine and Tryptophan residues, which are diagnostic markers of protein side chain environment, and on the Amide I band, of which the deconvolution allows us to extract information about α-helix, β-sheet, and random coil contents. The results show an increase of the secondary structure content of DBD concomitantly with a decrease of its conformational heterogeneity upon its binding to Azurin. These findings suggest an Azurin-induced conformational change of DBD structure with possible implications for p53 functionality.

## 1. Introduction

p53 is an important tumor suppressor protein working as a central hub in a complex interaction network in which it regulates numerous cellular processes, including cell cycle progression, apoptosis induction, and DNA repair [1,2]. p53 is a member of the important class of intrinsically disordered proteins (IDPs), possessing both structured and disordered domains under physiological conditions and different conformations coexisting in solution [3]. Such a structural plasticity confers to IDP an extremely high conformational adaptability, allowing them to act according to functional modes not achievable by ordered proteins, with these properties having been recently exploited to develop engineered protein and peptide drugs [4,5,6]. 

p53 is a tetrameric protein composed of four identical subunits and acts as a transcription factor. Each monomer of p53 consists of an N-terminal transactivation domain (NTD), a C-terminal domain (CTD), and a core DNA-binding domain (DBD) [7,8,9,10] The presence of unstructured portions allows p53 to adopt widely different conformations, which are at the basis of a vast repertoire of available interactions to different biological partners [11]. Among them, Azurin (AZ), a copper-containing electron-transfer anticancer protein secreted by *Pseudomonas aeruginosa* bacteria, has demonstrated the ability to specifically bind to p53, leading both to its stabilization and to an intracellular level increase both in vitro and in vivo [12,13,14,15,16,17]. Therefore, the formation of the p53-AZ complex has opened new perspectives in cancer treatment, such as the development of an AZ-derived anticancer peptide [18]. 

Keeping in mind the crucial role of AZ in assisting the oncosuppressive function of p53, in our group, we investigated the interaction between p53 and AZ at the single molecule level by Atomic Force Microscopy (AFM) and Atomic Force Spectroscopy (AFS) and by computational approaches [12,19,20,21]. These studies have provided information about the interaction kinetics between p53 or its DBD and AZ, obtaining also some relevant insights on the possible binding sites [21]. However, no experimental evidences on possible structural alterations of p53 upon its binding to AZ are so far available [3]. In this respect, Raman spectroscopy represents a suitable approach to extract information about the secondary structure of proteins as well as to probe their conformational heterogeneity, including IDPs [22]. Indeed, we have previously applied such a technique to investigate the structure and the conformational heterogeneity of wild-type and mutants p53 and, also, of the AZ-derived anticancer p28 peptide, even in different environmental conditions [18,23,24]. 

In the present work, we have employed a Raman-based approach to investigate if and how the native conformation of DBD is modified by its interaction with AZ. To such an aim, we have focused on an accurate inspection of the Fermi doublets relative to Tyrosine (830 and 850 cm^−1^; Tyr) and of Tryptophan peaks (1340 and 1360 cm^−1^), with these Raman signals having been recognized as suitable diagnostic markers of protein side chain environment [25,26]. Additionally, we have investigated the Amide I Raman band (1600–1700 cm^−1^), of which the deconvolution has demonstrated to be particularly effective in both extracting conformational information (α-helix, β-sheet, and random coil motifs) and which is a reliable reporter on the structural heterogeneity of proteins [22,27,28,29,30,31,32]. The Raman spectra have also been analyzed by applying principal component analysis (PCA), which performs a dimensionality reduction of the spectra, allowing a revelation of the differences between the complex Raman spectra of the samples and helping to understand the principal factors affecting the spectral variation [33]. 

The combination of these approaches has put into evidence the occurrence of structural changes within p53DBD upon its interaction with AZ. In particular, passing from isolated DBD to DBD bound to AZ, we found a variation in Tyrosine (Tyr) and Tryptophan (Trp) residues hydrophobicity and an increase of the DBD secondary structure concomitantly with a significant reduction of the conformational heterogeneity. The observed changes in both the structure and conformational heterogeneity of DBD strongly support the ability of AZ to modulate the DBD structure, and this, in turn, may result in a stabilization of the oncosuppressive function of p53.

## 2. Results and Discussion

### 2.1. Raman Analysis of AZ and DBD

**Figure 1:**
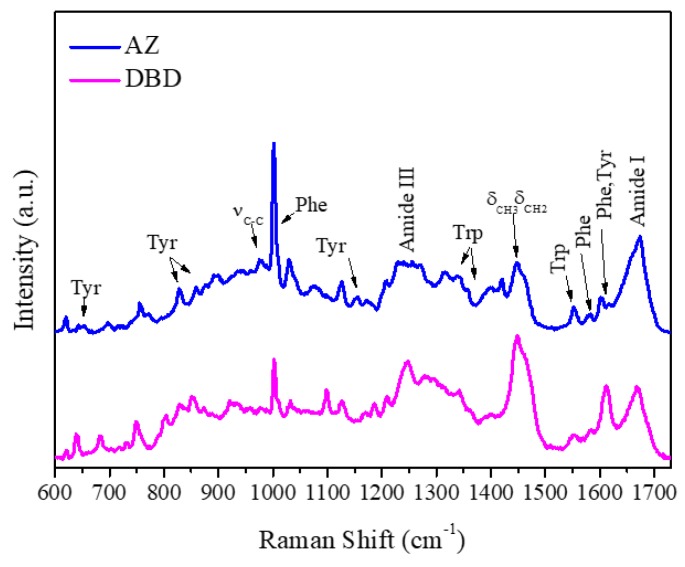
Raman spectra (600–1730 cm^−1^) with excitation at 532 nm of Azurin (AZ; blue) and DNA-binding domain (DBD; magenta) in Phosphate Buffer Solution (PBS): The principal proteins’ vibrational modes are marked. Spectra were normalized in the all spectrum frequency region and baseline corrected for a better visualization.

Figure 1 shows the Raman spectra of AZ and DBD in the 600–1725 cm^−1^ frequency range. The spectra display a complex set of bands arising from the modes of the aromatic amino acids (Tyr, Trp, and Phenylalanine (Phe) and of the peptide backbone, consistent with the typical Raman spectra of proteins [27,34]. The assignments of the main peaks are summarized in Table 1 [27].

Among the main Raman markers, we focused our attention on the Raman peaks of Tyr and Trp residues, which allow the extraction of information on protein side-chain local environment and on the Raman band of Amide I, which provides a diagnostic of the protein secondary structure. 

**Figure 2:**
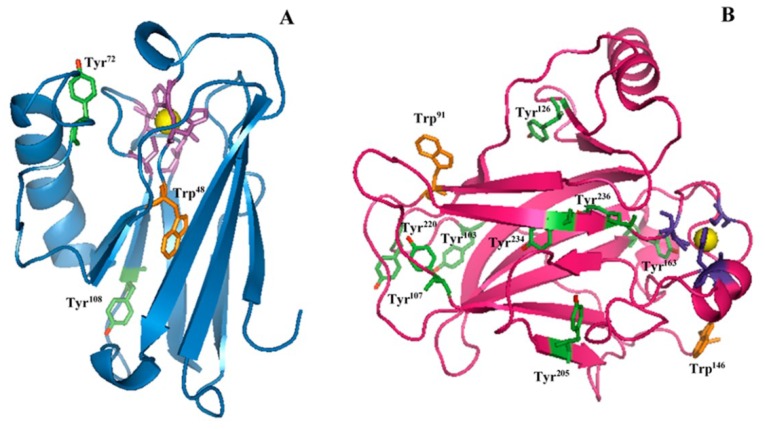
Three-dimensional structures of (**A**) AZ (PDB code: 4AZU) and (**B**) DBD (PDB code: 2XWR): The active site of AZ and the zinc-finger of the DBD are shown as yellow ball and stick models. The aromatic residues are Tyr (green) and Trp (orange). The OH groups in Tyr residues are marked in red.

Concerning the Tyr residues, the ratio I_Y_ = I_850_/I_830_ between the intensity of doublet peaks at 850 and 830 cm^−1^ is related to the donor or acceptor role of the Tyr phenoxyl group. Specifically, a low I_Y_ value (around 0.3) indicates the phenolic hydroxyl (OH) group acting as a strong hydrogen bond donor, as occurring for buried tyrosine residues. As the I_Y_ value increases (until 2.5), the phenolic oxygen becomes a stronger hydrogen bond acceptor, while a largely enhanced value (I_Y_ > 6.7) represents a non-hydrogen-bonded state [25,26]. Experimental results on isolated AZ reveal an I_Y_ value of 0.38 ± 0.07, representative of a buried environment for its two Tyr residues (Tyr^72^ and Tyr^108^) in agreement with the X-ray structure of AZ, in which Tyr^72^ belongs to the peripheral α-helix region with a moderate solvent accessibility and Tyr^108^ is practically inaccessible to solvent (see Figure 2A) [35].

Isolated DBD exhibits an I_Y_ ratio of 1.37 ± 0.16, indicating a predominant exposition to the solvent surfaces of the eight Tyr residues. From X-ray structure, the phenolic OH groups of Tyr^103^ and Tyr^107^ are highly oriented towards the solvent (see Figure 2B) [7]. Moreover, Tyr^126^ and Tyr^205^, located at a crucial protein region interfacing with the DNA, show moderate accessibility, similar to that of Tyr^220^ located on the surface of the protein [7,8]. Finally, the remaining Tyr^163^, Tyr^234^, and Tyr^236^ are almost inaccessible to the solvent [8]. Therefore, our results are consistent with the X-ray data endorsing the DBD–Tyr high solvent exposition. 

Further information about the side chains can be achieved by analyzing the Fermi doublet bands of Trp residues at 1340 and 1360 cm^−1^, which are reporters of the hydrophobicity/hydrophilicity neighboring the Trp indole ring [32]. In particular, an intensity ratio I_W_ = I_1360_/I_1340_ smaller than 1.0 reflects a hydrophilic environment, while a ratio greater than 1.0 indicates a hydrophobic one [9,32]. 

For AZ, we found an I_W_ ratio of 1.54 ± 0.10, indicative of a buried and solvent inaccessible environment for the lone Trp residue. This is in accordance with the AZ X-ray data, showing that the Trp^48^ is deeply embedded in a highly hydrophobic core and surrounded by a closely packed β barrel structure (Figure 2A) [35]. 

We found for DBD an I_W_ ratio of 0.68 ± 0.10, which implies, on average, a moderate hydrophilic environment for its Trp^91^ and Trp^146^. The latter is positioned in a hydrophobic side chain and oriented towards the solvent, while the former is located at the N-terminus of DBD and displays a high solvent accessibility, as it comes out from the X-ray data (Figure 2B) [36]. However, Trp^91^ has been shown to be crucially involved in the packing process of DBD through interaction with the Arg^174^ residue, which reduces its solvent exposure [36]. Therefore, our data suggest that both Trp residues in DBD globally experience a hydrophilic environment. 

Information on protein secondary structure can be extracted by the Amide I band (1600–1700 cm^−1^), mainly arising from C=O stretching and the combination of the C–N stretching, the Cα-C–N bending, and the N–H in-plane bending modes of peptide group. Such a band is usually used as a marker for secondary structure components. In particular, when Amide I band is centered at 1655 cm^−1^, it indicates a prevailing α-helix conformational arrangement, while a shift of this band peak toward 1670 cm^−1^ is indicative of β-sheet conformation [22]. On the other hand, an analysis of the Amide I shape means an appropriate deconvolution strategy allows for the quantification of the percentage content of secondary structure components present in the protein [22,28]. 

**Figure 3:**
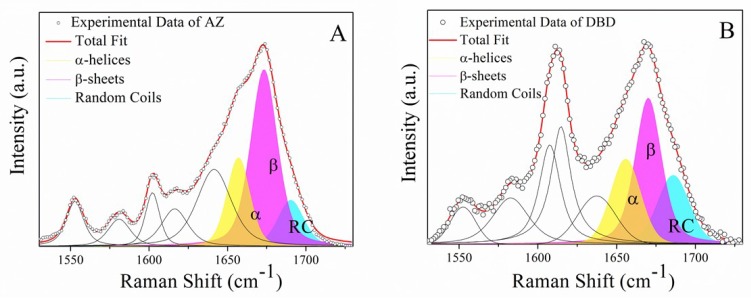
The Amide I band of AZ (**A**) and DBD (**B**) in PBS: The percentage of secondary structure for these proteins has been estimated from the relative area of deconvoluted bands of this spectral region of which the fitting parameters are reported in Table 2.

Specifically, the Amide I band of AZ emerges at about 1670 cm^−1^ (Figure 3A), suggesting a predominant β-sheet conformation [37]. The curve-fitting procedure points out β-sheet conformations predominant for 60%, while the α-helices and random coils account for about 22% and 18%, respectively. The obtained AZ secondary structure is agreement with that determined by X-ray diffraction for the crystals of AZ. Indeed, the major AZ components are β strands and turns (≈69%), which form two sheets arranged in a Greek key motif and with a minor contribution from a rigid α-helix (about 31%), conferring to AZ a low level of flexibility and structural disorder (see Figure 3A) [21,35,38]. 

Concerning DBD (Figure 3B), the band corresponding to the β-structures provides 46% of the total, while those related to α-helix and to random coils have 25% and 29%, respectively. These results indicate that DBD is characterized by a partially ordered structure, combined with the presence of significant disordered regions. Additionally, the results confirm those reported in our recent study on different sample batches of DBD (aminoacids 81–300), from which a content of 27% and 50% for α-helical and β conformations, respectively, have been estimated [23]. Moreover, these data are in agreement with X-ray data indicating a 30% of β-arrangement with an 18% of α-structures (see Figure 3B) [36]. The DBD propensity to adopt a predominant β-conformation is actually related to the large presence in its sequence of hydrophobic residues, such as Cysteine (Cys), Trp, and Leucine (Leu), generally promoting an ordered structure [39].

### 2.2. Raman Analysis of the DBD:AZ Complex

**Figure 4:**
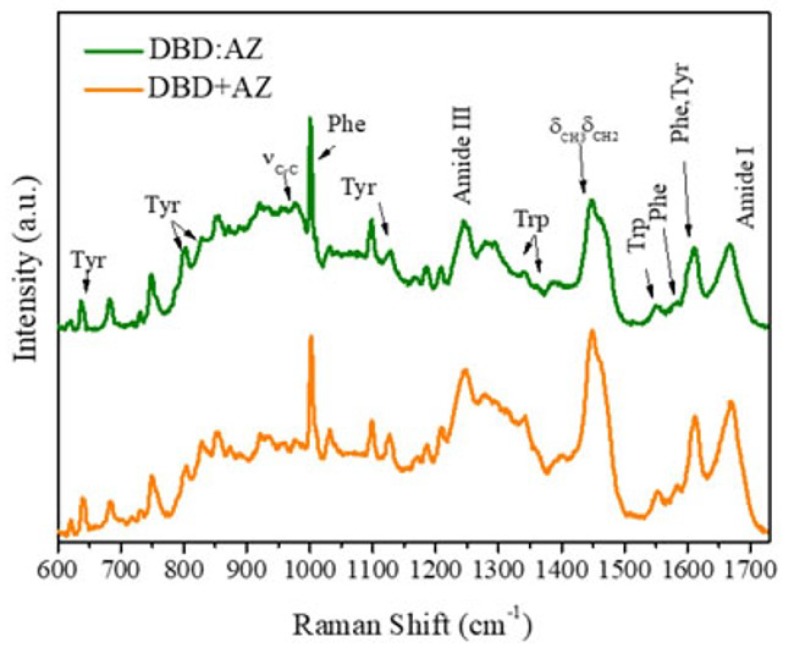
Comparison among the 532-nm-excited Raman spectra (600–1730 cm^−1^) of DBD:AZ complex (green) and of added spectrum AS (orange) in PBS: The principal proteins’ vibrational modes are marked. Spectra were normalized in the all spectrum frequency region and baseline corrected for a better visualization.

The previous analysis on the Raman spectra of AZ and DBD proteins, isolated in solution, has provided information on their structural properties paving the way to investigate possible structural change when they are involved in the formation of a complex. The spectrum of DBD:AZ solution, obtained by mixing equimolar amounts of DBD and AZ in the 600–1725 cm^−1^ frequency region is shown in Figure 4. We note almost the same general features displayed by the isolated protein spectra with no significant shifts in frequency for the main vibrational modes. From the Tyr peaks visible at 828 and 854 cm^−1^, the Fermi doublet ratio I_Y_ is 0.58 ± 0.08, which is indicative of a predominant hydrophobic environment. Such a value is closer to that of AZ (I_Y_ = 0.38 ± 0.07) with respect to that of DBD (I_Y_ = 1.37 ± 0.16), suggesting some changes in the Tyr microenvironment resulting from the interaction between the two biomolecules. To further support such a hypothesis, we have analyzed the Raman spectrum obtained by directly summing the spectra of isolated DBD and AZ molecules acquired at the same concentration used to form the complex (Figure 4), with the resulting spectrum being called added spectrum (AS) in the following. The analysis of the Tyr peaks in the AS spectrum reveals an I_Y_ of 1.11 ± 0.18, which is indicative of an average hydrophilic environment for all the Tyr residues in the system, as expected for isolated proteins. Therefore, the marked differences between the I_Y_ values from the complex and AS spectra can be ascribed to changes due to the interaction between the molecules. Although the spectroscopic results alone cannot allow us to identify the Tyrs that are involved in the structural changes, they support literature data that point out the involvement of the S_7_–S_8_ loops, comprising Tyr^220^, Tyr^234^, and Tyr^236^ and also Tyr^126^ at DBD binding sites with AZ, which, in turn, is engaged through its a.a 50–77 fragment, including Tyr^72^ [40]. 

The spectrum of DBD:AZ shows that the Trp Raman peaks are located at the same frequencies as in the isolated proteins, with an I_W_ ratio of 1.15 ± 0.17, indicating a high hydrophobicity for the three Trps residues (AZ–Trp^48^ and DBD–Trp^91^/Trp^146^). The found value of I_W_ for DBD:AZ, slightly lower than that for AZ (I_W_ = 1.54 ± 0.10) and higher than both isolated DBD (I_W_ = 0.68 ± 0.10) and AS (I_W_ = 0.53 ± 0.10), suggests some modifications in the environment experienced by these residues upon complex formation. The AZ–Trp^48^ is well-known to be strongly buried in the central hydrophobic core of the AZ; therefore, these changes can be due to variations in the DBD–Trp neighboring. Additionally, since the DBD–Trp^91^ has been shown to be engaged with the Arg^174^ [36], we suggest that the observed modifications of DBD as due to AZ interactions occurring within the DBD–Trp^146^ environment, with this being in agreement with Docking and Molecular Dynamics (MD) data showing the involvement of Trp^146^ in AZ-binding site [40]. 

**Figure 5:**
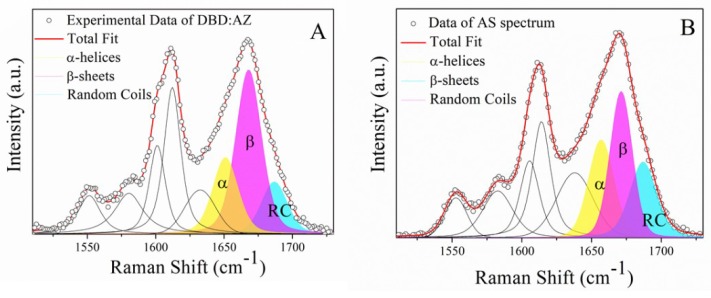
Amide I Raman band (open circles) excited at 532 nm of (**A**) DBD:AZ and (**B**) AS spectra, fitted through the Levenberg–Marquardt minimization algorithm (LMA; red line) in which the AZ total fit has been imposed as a constraint: Solid curves indicate the main structural conformations (α-helices, β-sheets, and random coils). Fitting results are summarized in Table 2.

Figure 5A,B shows the fitted curves of the Amide I band for the DBD:AZ and AS spectra, respectively. The results of the best fit for both experimental and AS Amide I bands, obtained by applying the same method used for isolated molecules, are reported in Table 2. DBD:AZ shows a predominant contribution from β-sheet structures (51%) and an α-helix amount of about 26%, while the random coil conformations contributes to 23% of the total Amide I band area. Best fit of AS reveals a predominant β structure (41%) with α-helices and random coils percentages of 31% and 28%, respectively. The observed changes in the secondary structure composition in DBD:AZ with respect to those of DBD and AZ can be attributed to the interaction between these proteins. Furthermore, since AZ is characterized by a highly structured conformation, the decrease of random coil structures can be mainly attributed to DBD. Such a result is supported by previously reported molecular dynamics simulations and docking studies showing that the binding of AZ at the peripheral, unstable, L_1_ and S_7_–S_8_ loops of DBD can enhance their stability upon restraining their flexibility [21], with this being in agreement with a reduction of the DBD disordered regions upon binding to AZ. Accordingly, it could be hypothesized that the increase of structural stability of DBD could be at the basis of the anticancer effect exerted by AZ. Since the structural dynamics and the interactions between proteins are strictly connected, a deeper characterization of the structural–functional relations is of fundamental interest for developing AZ-based drugs, of which effective action in vivo requires, however, further validations [41].

### 2.3. Principal Component Analysis of DBD, AZ, and the DBD:AZ Complex

**Figure 6:**
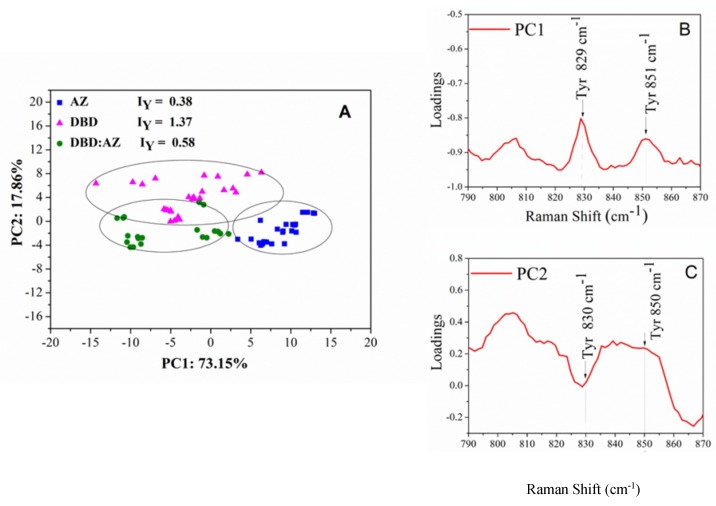
(**A**) Two-dimensional scores plot PC1 versus PC2 of the Raman spectra for AZ (blue squares), DBD (magenta triangles), and DBD:AZ complex (green circles) in the PBS performed on Fermi Doublet of Tyr region (790–870 cm^−1^): The three groupings are indicated by ellipses. The Fermi Doublet ratio for Tyr residues are also reported. (**B**) PC1 (73% of total variance) and (**C**) PC2 (18% of total variance) one-dimensional loadings plot versus frequency. The Raman markers are indicated.

Different combinations of scores for the first three principal components (PC1, PC2, and PC3) have been used to build two-dimensional plots; in the following, the components releasing the highest structural information will be shown. Figure 6A shows the PCA scores of PC1 vs. PC2 components (providing about the 90% of the total variance) for the Fermi Doublet region relative to Tyr residues (790–870 cm^−1^) for the AZ and DBD isolated molecules and for the DBD:AZ complex. In the scatter plot, two distinct groupings along the PC1 axis can be identified (see the ellipses drawn as a guide). Indeed, the AZ scores (blue symbols) are located in the positive portion of the plot along PC1 with a low spread along both the axes (10 and 5 along PC1 and PC2, respectively), while the DBD and DBD:AZ scores (magenta and green symbols, respectively) occupy the negative range of PC1 values. Along PC1, a larger variability is detected for DBD with respect to AZ and DBD:AZ. Additionally, along PC2, for AZ and DBD, negative values of PC2 are obtained while positive values are detected for DBD. To correlate the position of the scores in the plot with the samples’ spectral features, we have analyzed the loadings with the variables mostly contributing to the PCA scores. As shown in Figure 6B,C, high levels of variance are detected in correspondence with the peaks at 829 cm^−1^ and 851 cm^−1^ related to the Fermi Doublet of Tyr modes and with a weaker peak at 805 cm^−1^, associated with Tyr [25,32,42]; the latter provides the largest variance for PC2 loadings (see Figure 6B) [42]. These results show that Tyr vibrational modes are responsible for sample differentiation in PCA, consistent with our previous study supporting the important role played by Tyr modes as structural markers [25,42].

**Figure 7:**
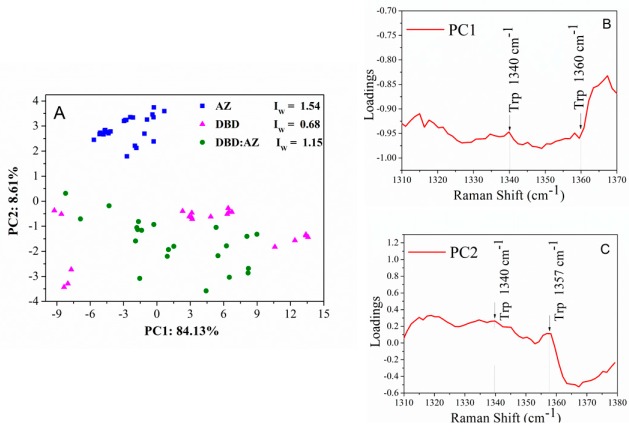
(**A**) Two-dimensional scores plot PC1 versus PC2 of the Raman spectra for AZ (blue squares), DBD (magenta triangles), and DBD:AZ complex (green circles) in the PBS performed on Fermi Doublet of Trp region (1310–1380 cm^−^^1^): The Fermi Doublet ratio for Tyr residues are also reported. (**B**) PC1 (84% of total variance) and (**C**) PC2 (8% of total variance) one-dimensional loadings plot versus frequency. The Raman markers are indicated.

**Figure 8:**
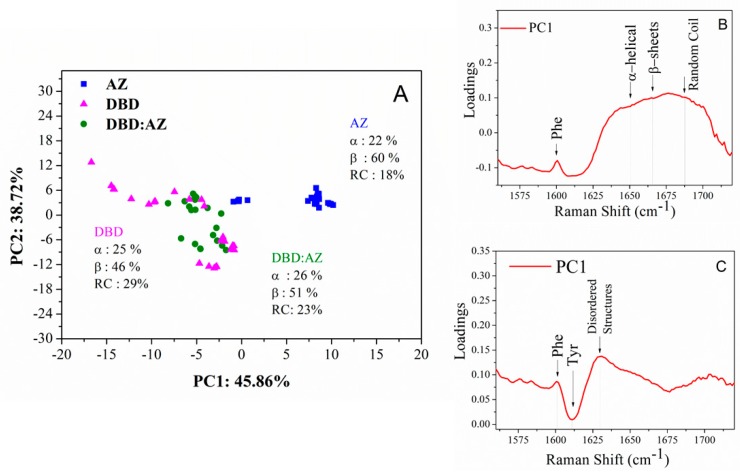
(**A**) Two-dimensional scores plot PC1 versus PC2 of the Raman spectra for AZ (blue squares), DBD (magenta triangles), and DBD:AZ complex (green circles) in the PBS performed on the Amide I band (1560–1720 cm^−1^): The secondary structure percentages as obtained by the curve fitting analysis are also reported. (**B**) PC1 (46% of total variance) and (**C**) PC2 (39% of total variance) one-dimensional loadings plot versus frequency. The Raman markers are indicated.

We then applied PCA to the Fermi Doublet region relative to Trp residues (1310–1380 cm^−1^), with the PC1 and PC2 components providing about 91% of the total variance (see Figure 7A). AZ clusters at the upper side, DBD clusters at the middle, while DBD:AZ clusters at the lower region in correspondence to negative values of PC2 axis. Along PC1, DBD, and DBD:AZ, scores are mixed within an overlapped cloud, while AZ are well-clustered in a well-separated group. Concerning the loading plots, shown in Figure 8B,C, PC2 presents a broad band with a loading positive value of about 0.2. At 1360 cm^−1^, a single evident peak emerges as ascribed to one of the Fermi Doublet of the Trp modes. This suggests that the separation among the three groups along PC2 depends on Trp vibrational modes. Since such a frequency changes according to the different Trp side-chain environment taken into consideration [28], a different spatial arrangement of this residue should be envisaged in the DBD isolated molecule and in DBD:AZ complex. 

Finally, the PCA was performed on the Amide I Raman band of AZ, DBD, and the DBD:AZ complex. From the scatter plot of PC1 versus PC2 (see Figure 8A), AZ data (blue squares) cluster at the positive side of the PC1 axis, with a low variance along both of the components, while DBD (magenta triangles) and DBD:AZ data (green circles) are characterized by negative values of PC1, with some overlap between them. A significantly larger variability is detected in DBD with respect to that of DBD:AZ.

The PC1 loading curve, accounting for 46% of the variance (Figure 8B), is characterized by a very broad band from 1625 to 1730 cm^−1^, including all the frequencies related to the secondary structure of a protein. Such a band shows the lowest value for the 1650 cm^−1^ frequency and the highest for the 1680 cm^−1^ one, which are associated to the α-helices and disordered structures, respectively [19,24]. In PC2, accounting for a 39% of the variation in the spectra, the major source of variance comes from peak at 1627 cm^−1^, consistent with the disordered structures component (Figure 8C) [19]. This indicates that PC1 discriminates the data based on different amounts of secondary structure of the sample, while PC2 reflects the amount of conformational disorder. Indeed, the AZ scores are very close to each other, indicating a very low variability in the secondary structure within different batches of samples, with this being consistent with the AZ ordered secondary structure [32]. Additionally, the superposition of DBD and DBD:AZ data along PC1 can be explained by assuming that the intrinsic disordered nature of DBD is able to populate an ensemble of different conformations. On the other hand, DBD:AZ distribution on the plot is narrower than that of DBD, reflecting a lower degree of disorder in the complex. These results confirm that PCA is a good reporter of the different structural differences among AZ, DBD, and DBD:AZ. Moreover, PCA is sensitive to changes in the conformational heterogeneity of DBD in the presence of AZ.

## 3. Materials and Methods

### 3.1. Sample Preparation

AZ (purity > 80%; MW = 14.6 kDa) was purchased from Sigma–Aldrich (St. Louis, MO, USA). The effective purity of the sample was checked by determining the ratio of spectral absorption at 630 nm and at 280 nm. AZ batches with a ratio value higher than 0.48 were used, with this indicating a good degree of purity [43]. AZ was dissolved in MilliQ water at a concentration of 200 µM. DBD (a.a 89–293; MW= 23 kDa) was purchased from GenScript (Piscataway, NJ, USA). DBD were dissolved in Phosphate Buffer Solution (PBS; 95.3% H_2_O, 3.8% NaCl, 0.1% di KCl, 0.7% Na_2_HPO_4_, 0.1% KH_2_PO_4_; pH = 7.4), reaching a final concentration of 40 µM. The DBD:AZ complex in PBS solution were prepared by mixing equimolar amounts of the components. 

### 3.2. Raman Spectroscopy

Raman measurements were carried out using a Super Labram confocal spectrometer (Horiba, France), equipped with several objectives, a diode-pumped solid-state laser (532 nm) and a spectrograph, with an 1800 g/mm grating allowing a resolution of 5 cm^−1^. Raman spectra were collected by means of a liquid nitrogen-cooled charged coupled device (CCD) (back illuminated; pixel format: 1024 × 128 detector) and in the back-scattering geometry in which a notch filter was used to reject the elastic contribution. All the experiments were performed using a laser power of 10 mW (4.4 mW on the sample) and a 50× objective with a numerical aperture NA = 0.6 (laser spot diameter reaching the sample was about 1 μm). A large confocal diaphragm (400 μm) and a slit of 200 μm were used to obtain a good Raman signal. 

Protein drops (10–15 µL) were deposited onto an optical glass, and spectra were acquired on partially dried samples. Indeed, it was demonstrated that there are no significant differences between the Raman spectra of protein in solution and the corresponding drop coating deposition, in which the protein remains substantially hydrated and the secondary structure is largely preserved [44].

Each Raman spectrum was acquired at room temperature by averaging 10 scans of 10 s integration time. For each sample, twenty-five Raman spectra were collected from different regions of the drops. Raman data processing and analysis were performed with OPUS software version 6.5 (Bruker Optics, Ettlingen, Germany). All the spectra were normalized with respect to the phenylalanine (Phe) ring breathing band at 1002 cm^−1^ due to its insensitivity to conformation or microenvironment [45], and the fluorescence background was removed by applying a rubber band baseline correction [46]. Finally, the spectra used for the structural analysis were obtained by averaging five measurements to improve the spectral signal/noise ratio. 

### 3.3. Analysis of the Raman Spectra

The secondary structure content of isolated DBD and AZ was quantified through a deconvolution procedure of the Amide I Raman bands by using three pseudo-Voigt profiles. The model parameters were optimized with the Levenberg–Marquardt minimization algorithm (LMA), and the goodness of the fit was assessed by the reduced chi-square value. The AZ curves as extracted from the fit were used in the DBD:AZ complex analysis, under the hypothesis that the AZ secondary structure does not change upon the interaction [38]. The three pseudo-Voigt profiles were centered at 1650–1656, 1664–1670, and 1680 cm^−1^ and assigned to α-helix, β-strand, and random coil conformations, respectively, as validated on other IDPs [22,23,27]. In each fitting analysis, additional peaks had to be included in the band-fitting protocol to account for aromatic residue modes (1550, 1580, 1604, and 1615 cm^−1^) and for disordered structure and/or vibronic coupling (1637 cm^−1^) not baseline separated from Amide I features [22]. The errors relative to secondary structure percentages were evaluated by repeating the curve-fitting procedure on five different spectra and the accuracy associated with the determined secondary structure content was about 10% for each sample.

In order to improve the performance of deconvolution analysis, we performed a dimensionality reduction of the Raman spectra based on principal component analysis (PCA) [33]. The PCA transforms the original data set into a new data set with transformed variables (principal components) that are linear combinations of the original variables. The principal components were arranged in a swat that the variability of the original data set was contained in descending order in the first principal components. PCA was applied to the isolated DBD and AZ molecules and to the DBD:AZ complex (number of spectra n = 75) in three different spectral regions: (i) Fermi doublets relative to Tyr (830 and 850 cm^−1^), satisfactorily described by a number of components N= 79; (ii) Fermi doublets relative to Trp (1340 and 1360 cm^−1^) described by N = 79; and (iii) Amide I Raman band (1600–1700 cm^−1^) described by N = 77. The number of components of the correlation matrix to be considered was defined as the number required to explain at least 80% of the total variance. STATISTICA 7.0 software (StatSoft Inc., Tulsa, OK, USA, 2004) was used for all the analyses.

## 4. Conclusions

The structural and conformational changes in the DBD region of the intrinsically disordered protein p53 upon interacting with the anticancer blue copper protein AZ were investigated by applying Raman spectroscopy. A careful inspection of the Raman spectra combined with a PCA analysis on the Fermi doublets of the Raman markers corresponding to the tyrosine and tryptophan residues allowed us to monitor the changes in their microenvironment as induced by the formation of a complex between DBD and AZ. Interestingly, we found a direct involvement of DBDTrp^146^ in the complex formation, as suggested by other experimental investigations. Additionally, a deconvolution of the Amide I band, remarkably sensitive to the α-helix, β-sheets, and random coil structures, allowed us to quantify the main secondary structural motifs of the DBD and its changes as induced upon binding to AZ. We found that DBD undergoes a slight increase of the β-conformation, with a concomitant lowering of its disordered portions as well as of its conformational heterogeneity. These findings are in agreement with our previous computational results and suggest that the binding of AZ to some unstructured motifs of DBD can restrain their flexibility. Collectively, the observed modulation the DBD structure when bound to AZ may represent a ground for understanding the molecular mechanisms of the AZ anticancer activity and could provide some hints for designing other molecules for p53-targeted therapies. Finally, we would remark that our Raman-based approach can be applied to investigate the structural changes of other biomolecules undergoing specific complex formation in order also to elucidate the molecular mechanisms which regulate their biological functions.

## Figures and Tables

**Table 1:** Typical proteins’ Raman vibrational modes (Raman cm^−1^) and related assignments.

Raman (cm^−1^)	Assignment
643	Tyr
805	Tyr
830,850	Tyr
870	Trp
902	ν_CC_
930,980	ν_CCN_
1001	Phe
1103	ν_CC,_ ν_CN,_ ν_CO_
1127	ν_CC_
1174	Tyr
1180	Phe
1210	Tyr
1230–1240	Amide III (α-helices)
1250–1255	Amide III (β-sheets)
1270–1300	Amide III (Random coils)
1320	CH_2_ deformation
1340,1360	Trp
1403	Symmetric ν_co2_^−^
1424	CH_2_, CH_3_ deformation
1451	CH_2_, CH_3_ deformation
1552	Trp
1604	Phe
1615	Tyr
1650–1680	Amide I

**Table 2:** Assignments, relative central frequency (Raman cm^−1^), and integrated intensities (Area %) of the main Amide I band components (α-helix, β-sheet, or random coil) for AZ, DBD, and DBD:AZ complex obtained by a fitting procedure. χ2 = 0.002 for all curve fitting analysis.

Sample In PBS	Secondary Structure	Raman Shift cm^−1^	Area (%)
**AZ**	α-helix	1659	22
	β-sheet	1674	60
	random coil	1688	18
**DBD**	α-helix	1655	25
	β-sheet	1670	46
	random coil	1686	29
**DBD:AZ**	α-helix	1655	26
	β-sheet	1669	51
	random coil	1687	23
